# National Child Passenger Safety Week — September 14–20, 2014

**Published:** 2014-09-12

In the United States, motor vehicle crashes are a leading cause of death among children (1). In 2012, a total of 1,168 passenger vehicle occupants aged 0–14 years died as a result of a motor vehicle traffic crash (2). During 1975–2012, child restraints saved an estimated 10,157 lives of children aged 0–4 years (2). Seating position also contributes to child passenger safety. To keep child passengers as safe as possible, drivers should properly restrain children aged <13 years in a back seat and follow the American Academy of Pediatrics’ child passenger safety recommendations, which include properly restraining children in age- and size-appropriate restraints as follows: rear-facing child safety seats up to age 2 years; forward-facing child safety seats up to at least age 5 years; booster seats through at least age 8 years and until seat belts fit properly; and adult seat belts, still in the back seat, until age 13 years. Passengers aged ≥13 years should use adult seat belts on every trip (3). Additional information on child passenger safety is available at http://www.cdc.gov/vitalsigns/childpassengersafety/index.html.

For 2014, National Child Passenger Safety Week is September 14–20. As part of the campaign, September 20 is designated as National Seat Check Saturday, when drivers with child passengers are encouraged to visit a child safety seat inspection station to have a certified technician inspect their car seat and give hands-on advice free of charge. Additional information and an inspection station locator are available from the National Highway Traffic Safety Administration at http://www.safercar.gov/cpsApp/cps/index.htm. Promotional materials (in English and Spanish) are available at http://www.trafficsafetymarketing.gov/cps.
